# Dermoscopy of skin infestations and infections (entomodermoscopy) – Part II: viral, fungal and other infections^☆^^☆☆^

**DOI:** 10.1016/j.abd.2021.04.008

**Published:** 2021-10-05

**Authors:** Renato Marchiori Bakos, Leandro Linhares Leite, Clarissa Reinehr, Gabriela Fortes Escobar

**Affiliations:** a Universidade Federal do Rio Grande do Sul, Porto Alegre, RS, Brazil; b Dermatology Service, Hospital São Lucas, Pontifícia Universidade Católica do Rio Grande do Sul, Porto Alegre, RS, Brazil; c Postgraduation in Medical Sciences, Universidade Federal do Rio Grande do Sul, Porto Alegre, RS, Brazil; d Hospital de Clínicas de Porto Alegre, Porto Alegre, RS, Brazil

**Keywords:** Dermatomycoses, Dermoscopy, Skin diseases, infectious, Skin diseases, viral

## Abstract

In addition to the infestations and bacterial infections reported in part I, the study of entomodermoscopy also involves descriptions of dermoscopic findings of a growing number of viral and fungal infections, among others. In this article, the main clinical situations in viral infections where dermoscopy can be useful will be described, that is in the evaluation of viral warts, molluscum contagiosum, and even in recent scenarios such as the COVID-19 pandemic. As for fungal infections, dermoscopy is particularly important, not only in the evaluation of the skin surface, but also of skin annexes, such as hairs and nails. The differential diagnosis with skin tumors, especially melanomas, can be facilitated by dermoscopy, especially in the evaluation of cases of verruca plantaris, onychomycosis and tinea nigra.

## Introduction

In addition to the usefulness of dermoscopy for the evaluation and detection of dermatozoonoses and several bacterial infections, it can be of great help in the evaluation of different viral, fungal (superficial and deep) infections and other infectious diseases. Moreover, follow-up with dermoscopy can be useful to analyze the therapeutic efficacy in some of these diseases. In part II of this review, the authors of the present study will emphasize the main clinical situations caused by viral, fungal and other infections where dermoscopy can be useful.

## Viral infections

### Verrucae

Warts are skin infections caused by the human papillomavirus (HPV), transmitted through direct contact with infected skin or mucosa or through fomites. According to their anatomical or morphological location, they can be classified into common, flat, anogenital (condyloma), or palmoplantar warts. Dermoscopy is an important tofool for the diagnosis and monitoring of the treatment of warts.^1^, ^2^

#### Common warts (verruca vulgaris)

**Figure 1:**
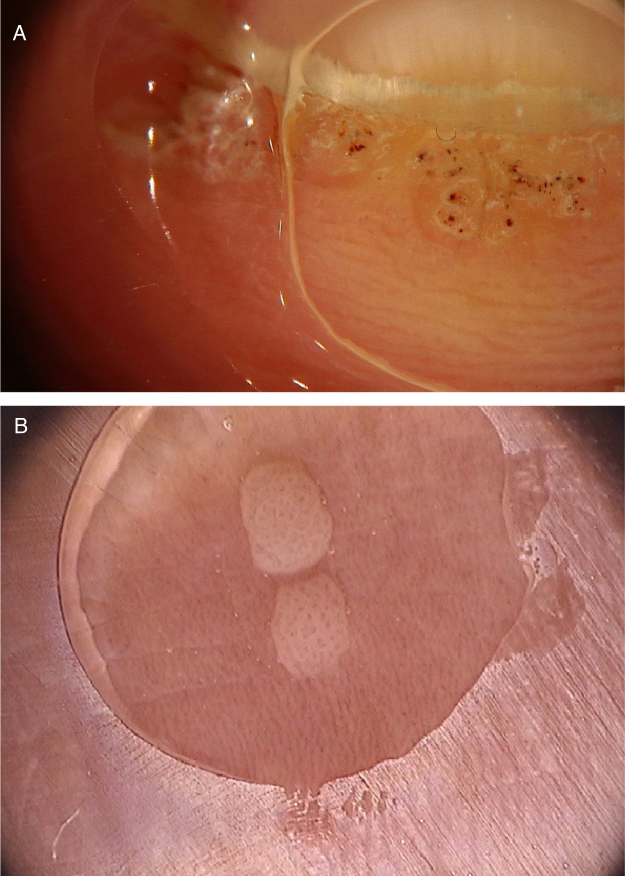
(A), Dermoscopic image of a verruca vulgaris in the subungual region showing hyperkeratosis and dotted or thrombosed vessels; (B), Dermoscopic image of a verruca plana showing discrete homogeneously distributed dotted vessels. (FotoFinder, original magnitude ×20). Source: Authors' personal collection.

Common warts are clinically characterized by exophytic papules with a rough, papillomatous surface. The most frequently affected sites include the hands and fingers. Moreover, pedunculated and filiform lesions can be observed, especially in the periorificial facial regions. On dermoscopic examination, papillomatous areas with thrombosed vessels can be seen in the center of each papilla (Fig. 1A). These findings have been described in the literature as a “frogspawn” pattern, which shows multiple polyps resembling a mass of frog eggs.^3^

#### Flat warts (verruca plana)

Flat warts are characterized by the presence of normochromic pinkish or brown papules, with a flat, smooth surface. They can be most frequently seen on the back of the hands, upper limbs or face. Dermoscopy reveals dotted or globular vessels with regular distribution on a yellowish-brown background (Fig. 1B).^4^ The histopathological correspondence of the dotted vessels is the apex of the capillaries in the papillary dermis.^3^ One of the main differential diagnoses of flat warts comprises small or initial seborrheic keratoses. In addition to the classic dermoscopic pattern, other clinical factors that help in the diagnosis of flat warts are the observation of lesions with a linear distribution (Koebner's phenomenon) or in a cluster and the absence of dermoscopic criteria for the diagnosis of seborrheic keratosis, such as the cerebriform appearance.^4^

#### Genital warts

Anogenital warts, or condylomas, affect the perineal, perianal or inguinal region. They are sessile lesions, varying from brownish to white (when found in areas subjected to maceration). Additionally, pedunculated or papillomatous lesions can be observed, which have a “cauliflower” pattern.

Some patterns have been described on dermoscopy for genital warts.^5^ The first, the mosaic pattern, refers to regular, clustered white rounded structures that form a reticular structure with central islets of healthy mucosa.^3^ This pattern is most often associated with flat genital lesions. The second pattern, called “knoblike,” refers to clustered bulbous projections of similar diameter and length. Finally, the third pattern is called “fingerlike” and is characterized by separate fingerlike projections, with different lengths. These two patterns can be seen in more exophytic and papillomatous lesions. The most common vascular structures include glomerular, dotted, and hairpin vessels. The first two are most commonly seen in the mosaic and knoblike patterns, whereas hairpin vessels are more often seen in the finger-like pattern. Other dermoscopic findings include the presence of pigmentation and hyperkeratosis. Dermoscopy can also aid in the differential diagnosis with physiological genital findings, such as pearly penile papules, Fordyce spots, and vestibular papillae. Penile pearly papules are angiofibromas regularly distributed in the crown of the glans which on dermoscopy show white-pink papules in a ‘cobblestone’ pattern, with dotted or comma-shaped vessels in the center of the papules. In the female genitalia, on the other hand, the vestibular papillae show multiple pink cylindrical projections, with a soft consistency and projection bases separate and symmetrically distributed.^6^

#### Palmoplantar warts

Palmoplantar warts are endophytic, hyperkeratotic and often painful lesions. When they appear more superficially, with lesions that coalesce into large plaques, they are called mosaic warts or myrmecia.

**Figure 2:**
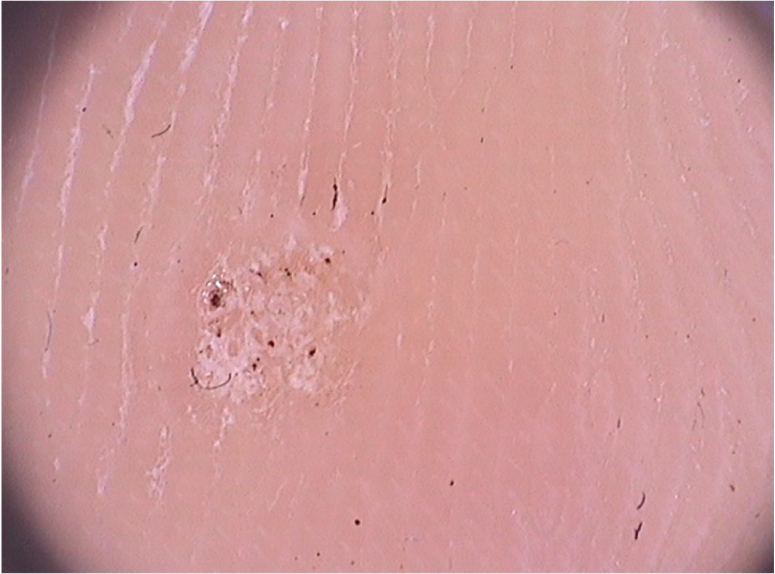
Dermoscopic image of a verruca plantaris with hyperkeratosis and thrombosed vessels. (FotoFinder, original magnitude ×20). Source: Authors' personal collection.

On dermoscopic examination, blackish dots with irregular distribution are seen on the surface of the wart, representing dilated and thrombosed capillaries (Fig. 2).^1^ Moreover, a hyperkeratotic or papillomatous surface can be seen, as well as the interruption of dermatoglyphics or microhemorrhages due to bodyweight pressure and distribution.

Dermoscopy is also an important tool to monitor the treatment of plantar warts, helping to detect the persistence of the lesion after partial treatment, even when they are clinically unapparent.^1^ Additionally, it also helps in the differential diagnosis with calluses and plantar keratoma, which show a homogeneous opacity or a translucent central nucleus.^1^ A noteworthy aspect is that cases of pigmented warts with a parallel ridge pattern have been described, which differential diagnosis includes acral melanoma. The opposite must also be remembered, i.e., hypopigmented or amelanotic acral melanomas can also mimic acral warts.^7^

### Epidermodysplasia verruciform

Epidermodysplasia verruciform is a rare autosomal recessive genodermatosis. It is characterized by a deficiency in the cellular immune response manifested by persistent HPV infection and a consequent propensity for the development of squamous cell carcinomas. Clinically, the appearance of flat macules and papules in childhood, affecting sun-exposed areas, is observed. The lesions may simulate seborrheic keratoses or pityriasis versicolor. Dermoscopic examination discloses the presence of a pinkish-brown or hypochromic background, with slight superficial desquamation, corresponding to lesions containing HPV-infected keratinocytes.^8^ Subsequently, other described findings included unfocused dotted vessels with a regular distribution and dilution of vellus hair pigment.^9^ Vessel proliferation, seen in other types of warts, is characteristic of HPV virus infection. In addition, it is questioned whether chronic HPV infection may interfere with melanogenesis, thus explaining the hypochromic lesions and dilution of hair pigment.^9^ These findings also help to differentiate keratinocytic neoplasms that occur in individuals with epidermodysplasia verruciform.

### Molluscum contagiosum

Molluscum contagiosum is a skin infection caused by the molluscum contagiosum virus (MCV), of the poxvirus family. It affects mainly children and is occasionally seen in sexually active adults and immunocompromised individuals. Its transmission occurs through direct contact with the infected skin, which also facilitates self-inoculation. It is clinically characterized by dome-shaped papules, pinkish-white in color, with a central umbilication. Atypical presentations, such as single or giant lesions, can also be observed, mimicking warts and epidermal cysts. Dermoscopy increases diagnostic accuracy when compared to clinical examination and also helps in the differential diagnosis of molluscum contagiosum with benign genital findings, such as pearly penile papules and Fordyce glands.^10^

**Figure 3:**
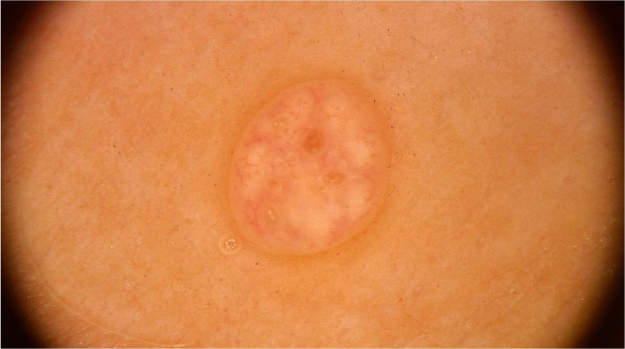
Dermoscopic image of molluscum contagiosum showing central umbilication, white-yellowish globular structures and predominantly peripherally distributed vessels. (FotoFinder, original magnitude ×20). Source: Collection of Hospital de Clinicas de Porto Alegre.

On dermoscopic examination, molluscum contagiosum often shows a central pore or umbilication, surrounded by a white-yellowish polylobular structure and peripheral vessels in a crown pattern (Fig. 3).^11^ Other vascular patterns that may be seen include radial, dotted, or a combination of the two former ones. When there is an association of crown and radial vessels, there is the so-called flower pattern, due to its resemblance to the petals of a flower. Moreover, the presence of dotted vessels has also been associated with inflammation in molluscum contagiosum, as in the case of excoriated lesions or with perilesional eczema.^10^ In turn, other variants of polylobulated structures include rounded structures (a white, discoid-like area) and the clover-like structure.^12^ Histopathological analysis shows that the lobules correspond to hyperplastic keratinocytes containing the typical intracytoplasmic viral inclusions that produce the peripheral displacement of the nucleus and are called Henderson-Patterson corpuscles. The variations in the dermoscopic features of the lobules may be explained by varying degrees of proliferation of the inverted lobes of the acanthotic epidermis.^12^

### Eruptive pseudoangiomatosis

Eruptive pseudoangiomatosis is a self-limited condition, characterized by the appearance of erythematous papules with a vasoconstriction halo. It is speculated that the lesions may be triggered by insect bites or viral conditions, including echovirus, Epstein-Barr virus, or cytomegalovirus. In these cases, viral symptoms (fever, cough, vomiting, and diarrhea) precede the lesions. Dermoscopy shows dotted vessels over a more prominent vascular network, which decreases significantly with vitropressure, associated with a vasoconstriction halo.^13^ The histopathological correspondence is ectasia, a mild perivascular lymphocytic infiltrate with intraluminal neutrophils and engorged endothelial cells.

### COVID-19

In late 2019, a new coronavirus emerged and spread rapidly, causing a pandemic. This virus was called severe acute respiratory syndrome coronavirus 2 (SARS-CoV-2) and has been associated with several dermatological manifestations, which include urticarial, morbilliform, vesicular, livedo reticularis, and acral ischemic lesions. To date, the main dermoscopic descriptions are of pernio-like erythema lesions.^14^

**Figure 4:**
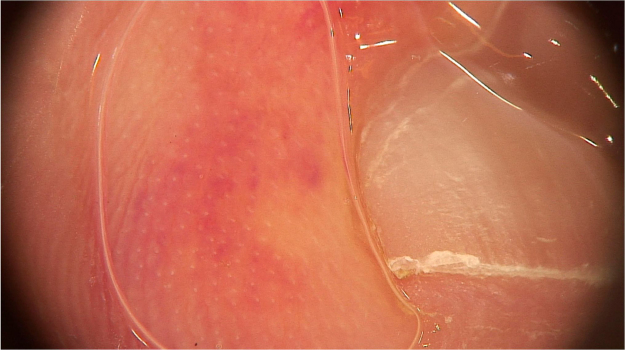
Dermoscopic image with erythematous-violaceous areas in a patient with perniosis-like erythema detected during the COVID-19 pandemic. (FotoFinder, original magnitude ×20). Source: Authors' personal collection.

Pernio-like erythema (chilblain-like eruptions) lesions affect mainly children, adolescents and young adults. They are typically characterized by macules or papules located in the acral regions, being erythematous-violaceous or purpuric in color. They may also present as erythematous-edematous areas, associated with pain or pruritus. The distribution of the lesions is usually asymmetric. They appear on average 14 days after the onset of a mild systemic condition and resolve after 7 to 10 days, being described as a late manifestation of the disease, when the PCR test can be negative.^14^ Dermoscopic examination shows an erythematous-violaceous background (Fig. 4), dilated capillaries, purpuric dots and, in the late phase, pigmented dots. The histopathological correspondence is a lymphocytic vasculopathy and it is questioned whether these cutaneous findings represent a coagulation disorder or a hypersensitivity reaction. Navarro et al. evaluated lesions at different stages of evolution.^15^ Initially, the lesions are erythematous due to vascular dilation, going through a stage of violaceous coloration due to the extravasation of red blood cells and, subsequently, a brownish hue, due to hemosiderin deposition. Grayish areas may indicate more intense ischemic phenomena. In addition, erythematous-violaceous globules and peripheral grayish-brown reticular areas were observed. The globules may represent areas of damaged vessels with extravasation of red blood cells, whereas reticular areas may result from significant damage to the dermal vascular plexus.

## Fungal infections

### Dermatophytoses

**Figure 5:**
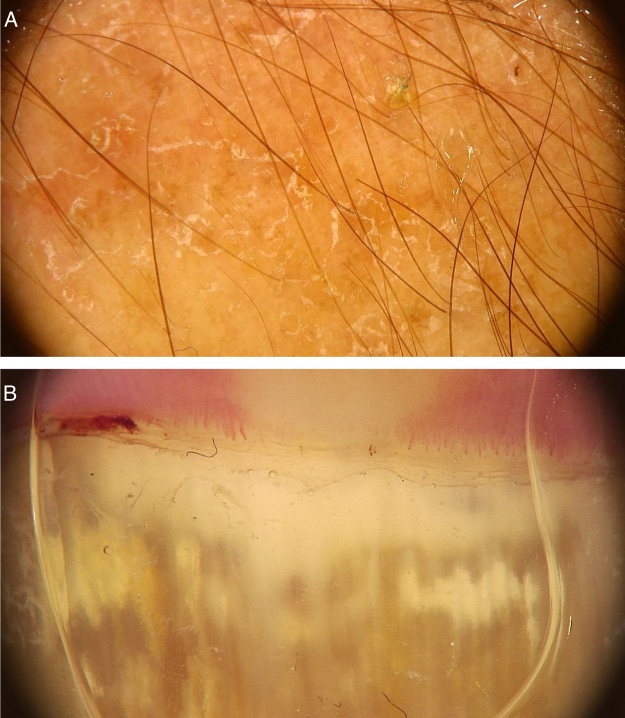
Dermoscopic image of tinea corporis showing areas of erythema and scaling (A) and of onychomycosis showing white-yellowish streaks in the proximal portion of the nail plate (B). (FotoFinder, original magnitude ×20). Source: Authors' personal collection.

Dermoscopic can be an auxiliary method to diagnose dermatophytoses. Onychomycosis, tinea capitis and tinea corporis are some of the dermatoses that can benefit from the use of dermoscopy for their diagnosis. In the case of onychomycosis, the capacity to differentiate it from traumatic onycholysis through dermoscopic findings stands out. The possibility of diagnosing cases of tinea incognita through dermoscopic findings has also been described (Fig. 5A).^16^ The main dermoscopic findings of the dermatophytoses are presented below.

### Onychomycosis

Onychomycosis represents 50% of all nail diseases, the worldwide of prevalence of which varies from 2% to 8%. It can be caused by different species: dermatophyte fungi, non-dermatophyte fungi, and leveduriform fungi.^17^ Approximately 90% of all hallux onychomycosis is caused by dermatophytes. The distribution of pathogens that cause this type of infection varies according to the geographic region, assessed population and climatic factors. Individual factors that predispose to the development of onychomycosis include diabetes, immunosuppression, venous insufficiency, peripheral artery disease, obesity, smoking, and older age.^18^ Additional factors include occupation, sports practice, wearing inappropriate footwear, inadequate nail trimming, tinea pedis, psoriasis, and a family history of onychomycosis.^18^ The most common age range for onychomycosis to occur varies from 4 0 to 6 0 years.^19^

The clinical aspects of onychomycosis are mainly onycholysis, changes in nail color, and subungual hyperkeratosis.^17^, ^18^ It can be divided into distal-lateral subungual (most common presentation), proximal subungual, superficial white, and total dystrophic onychomycosis.^18^

Although direct mycological examination and fungal culture are the gold standards for the diagnosis of onychomycosis, there are often limitations for these examinations. Dermoscopy can help its diagnosis and the exclusion of other onychopathies. A study of 50 patients (37 with distal subungual onychomycosis and 13 with onycholysis caused by trauma) observed some findings that were particular to distal-lateral subungual onychomycosis and others to onycholysis caused by trauma. Longitudinal streaks of different colors observed in the onycholytic nail plate (varying from white to yellow, orange, and even brownish in color) have been associated with distal subungual onychomycosis, being present in 86.5% (32/37) of the patients with onychomycosis and in none of the patients with traumatic onycholysis. The jagged edge with spikes, defined by the presence of whitish longitudinal indentations in the area of ​​onycholysis towards the proximal ungual edge, was observed in all cases of distal subungual onychomycosis evaluated in the study and in none of the patients with onycholysis caused by trauma. This finding would reflect the progression of dermatophytes in the stratum corneum of the ungual bed. Finally, the linear edge without indentations would only be seen in traumatic onycholysis.^20^ Other findings that can be observed on dermoscopy of the ungual plate with onychomycosis, but are not exclusive to this disease, include blackened dots and globules due to subungual hemorrhage and a dull and homogeneous color of the detached ungual plate, in which the color can vary from white to yellow, orange, brown and black, reflecting the color of the subungual colony, scaling, and debris.^20^ Another suggestive finding is the so-called “aurora pattern”, which refers to the fact that the onycholytic ungual plate has a dull, irregular color that is distributed in streaks, resembling the Northern Lights or aurora, found in distal subungual onychomycosis.^20^ The evaluation of onycholysis should also be performed by observing the proximal edge of the onycholysis area. If it is linear and without indentations, the possibility of onychomycosis is decreased, while the observation of a jagged edge with spikes and longitudinal streaks is highly suggestive for the diagnosis of onychomycosis (Fig. 5B).^20^

Other dermoscopic findings can be seen in onychomycosis, especially when other clinical presentations are evaluated. They include chromonychia, trachyonychia, yellowish-white longitudinal streaks, and proximal edges with indentations. In the lateral-distal subungual and total dystrophic subtypes, white longitudinal streaks and proximal edges with indentations were the most frequent findings.^19^ The presence of subungual hyperkeratosis seems to be more often observed in total dystrophic onychomycosis (p < 0.001) and leukonychia in proximal subungual onychomycosis.^5^, ^21^

Regarding melanonychia secondary to onychomycosis, some studies have attempted to differentiate it on dermoscopy from melanonychia of other causes, including ungual matrix nevi, ungual melanomas, and melanonychia due to melanocytic activation of the matrix.^22^ Ungual pigmentation due to fungal infection is not true melanonychia, as it does not originate from melanocytes, characteristically this type of pigmentation is non-longitudinal.^22^ Ohn et al. evaluated 80 patients with melanonychia from various causes, including 18 patients with fungal melanonychia, and observed that some findings were positive predictors of fungal melanonychia: association with yellowish color, homogeneous non-longitudinal pattern, reverse triangular pattern (in which the width of the pigmented area is greater in the distal portion than in the proximal portion of the nail, due to greater invasion of the nail plate by the fungus in the distal portion), subungual keratosis, and white or yellowish streaks or scaling.^22^ It is important to mention that the monitoring of onychomycoses with a pigmented presentation must have a frequent follow-up. Dermoscopy is of great help to determine the presence of other structures that suggest the possibility of a melanocytic collision lesion and also the response to treatment. In cases associated with melanonychia, improvement of ungual coloration is one of the earliest observed findings.^23^

### Tinea capitis

Tinea capitis is an infection caused by dermatophyte fungi that affect the skin of the scalp and hair shafts, which can be of the microsporic type, transmitted by animals, characterized by presenting a single plate; of the Trichosporon type, transmitted by interhuman contagion contact, which usually shows multiple lesions; of the favus type, or Kerion celsi type, an inflammatory form, with the presence of pustules and micro-abscesses. The disease most commonly affects children aged 3 to 7 years, but it may eventually affect adults. The increase in the prevalence of tinea capitis in recent decades and a change in the pattern of the dermatophytes that cause the disease have been observed in recent years.^24^ The etiological agents of tinea capitis vary according to the geographic region, climatic conditions and socioeconomic context of the population. Clinically, there are areas of hair loss, with tonsured hair shafts, associated with the presence of scaling, inflammation and pustules. Although direct mycological examination and culture are the gold standard in diagnosing this condition, they depend on the collection procedure and equipment to be performed.^25^ In this context, trichoscopy may help differentiate microsporic from Trichosporon infections and monitor the response to treatment.^25^

**Figure 6:**
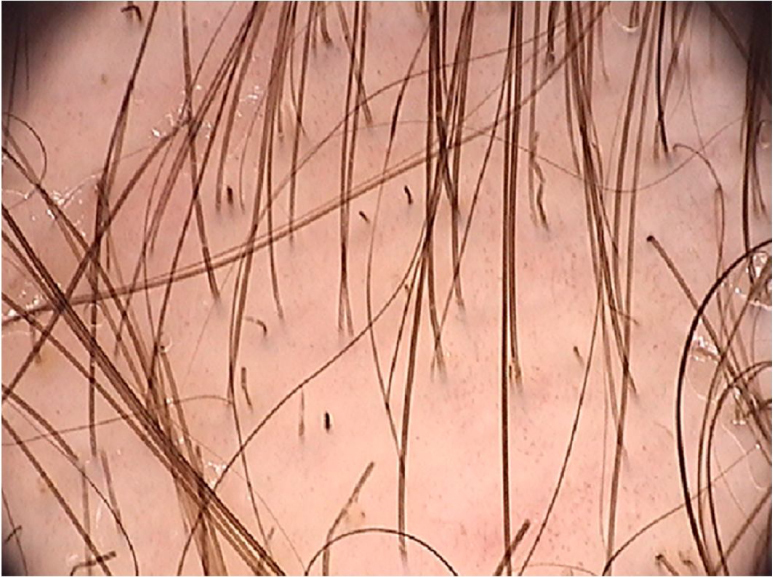
Dermoscopic image of tinea capitis showing broken and “comma-shaped” hairs (FotoFinder, original magnitude ×20). Source: Authors' personal collection.

A systematic review of the dermoscopic findings found in tinea capitis included 37 articles on the topic.^26^ Among the main findings, the “comma-shaped” hairs stand out, which are short hairs with homogeneous pigmentation and thickness, due to the breakage and twisting of the hair shaft filled with hyphae in its interior (Fig. 6).^27^ Their presence ranged from 13% to 100% in cases of tinea capitis, but they can also be seen in alopecia areata and trichotillomania.^26^ Corkscrew hairs, which consists of twisted hairs, are present in 14% to 100% (average of 32%) in cases of tinea capitis.^26^, ^27^ They are described as a specific finding of tinea capitis, seen with both endothrix and ectothrix fungi infections, although they may be seen in ectodermal dysplasias and in patients with vitamin C deficiency.^26^ “Morse code-like hairs” represent hairs with multiple thin whitish bands along the hair. They are formed by the accumulation of spores surrounding the hair shaft, causing a transverse perforation of the hair shaft, and are described as ectothrix-type infections, with an incidence of 12% to 56% (mean 22%).^26^ Zigzag-shaped hairs comprise hairs that are bent at sharp angles, the result of incomplete transverse fractures along the hair shaft. They are described in ectothrix-type fungal infections, with an incidence of 5% to 49% (mean 21%).^26^ In addition to being seen in tinea capitis, they can also be seen in alopecia areata.^27^ Among other findings that can also be seen on trichoscopy of tinea capitis, but which are not characteristic, broken hairs, black dots, and inter and perifollicular desquamation can be highlighted.^26^

Apparently, cases of tinea capitis caused by the genus *Microsporum* show more frequently “Morse code-like” hairs (8/29, 28%, p < 0.001), zigzag hairs (6/29, 21%, p < 0.01), folded hairs (4/29, 14%, p < 0.05) and diffuse desquamation (4/29, 14%, p < 0.05). These findings are correlated with ectothrix infections, i.e., the ones that occur around the follicular shaft, promoting transverse perforations of the hair shaft. Corkscrew hairs were more common in trichophytic cases (21/38, 55% vs. 3/29, 10% in microsporic cases; p < 0.001). In this case, the infection is of the endothrix type and, consequently, with hair shaft changes without changes in color.^26^

The disappearance of dystrophic hairs (comma-shaped, corkscrew, zigzag, Morse code, broken hairs, and black dots) can be an important trichoscopic parameter for evaluating therapeutic efficacy. These changes can take four to twelve weeks to disappear. Inter- and perifollicular scaling, on the other hand, take a longer period of time to resolve and thus should not be used to evaluate therapeutic failure.^26^

### Tinea nigra

**Figure 7:**
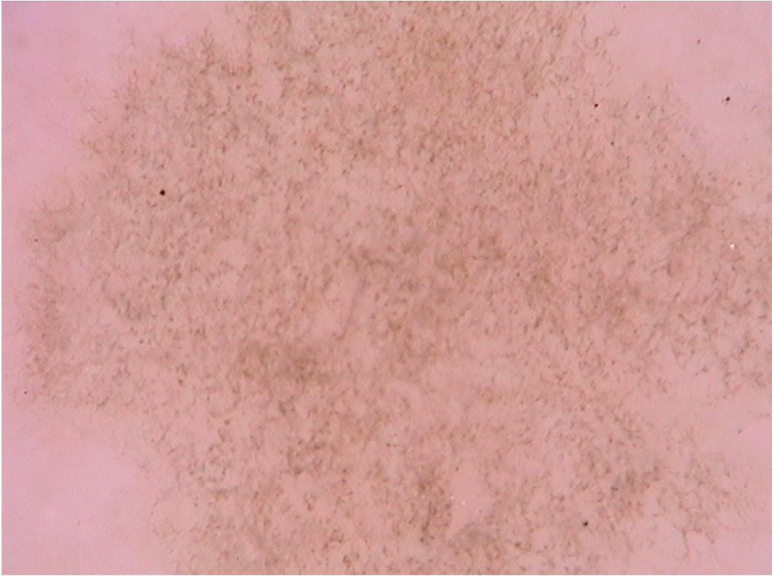
Dermoscopic image of tinea nigra demonstrating diffuse linear blackened structures. (FotoFinder, original magnitude ×20). Source: Collection of Hospital de Clinicas de Porto Alegre.

Tinea nigra is a superficial mycosis caused by the dematiaceous fungus *Hortaea werneckii*, which occurs predominantly in areas of tropical and subtropical climate. Clinically, it manifests as an irregularly pigmented brownish or blackish macula that classically occurs on the palms and soles. It usually shows progressive growth, which may be associated with scaling.^28^ An important differential diagnosis is made with melanocytic lesions, whether nevi or melanoma.^28^ Direct mycological examination reveals tortuous dematiaceous septate hyphae and the etiological agent is isolated in culture, confirming the diagnosis. Dermoscopic findings are the pigmented hyphae in the stratum corneum, with brownish pigment in spikes that form a reticulated pattern, without respecting the dermatoglyphic lines, in addition to the absence of a pigment network (Fig. 7).^29^ Other subsequent reports also demonstrated brownish pigment in small dots and granules over a lighter brownish macular area and again not respecting the dermatoglyphic ridges and sulci, as it would have been seen if this was a melanocytic lesion.^30^ Dermoscopy can help by avoiding unnecessary biopsies, as it allows differentiation with melanocytic lesions. It is also useful in evaluating therapeutic efficacy since the elimination of the pigmented lesion can be monitored by the technique.

### Pityriasis versicolor

Pityriasis versicolor is a superficial fungal infection caused by yeasts of the *Malassezia* genus, a lipophilic dimorphic fungus, which affects the superficial layers of the epidermis. Clinically, it shows hyper and hypopigmented round or oval scaling lesions, located on the trunk, upper limbs, and face, usually asymptomatic, although some patients report mild pruritus. There is a slight predominance of occurrence in males, and the age group with the highest occurrence is that of 11 to 20 years.^31^ In adults, it mainly affects the trunk, while in children it especially affects the face. These topographical differences result from variations in sebum production.^31^ Fungi of the *Malassezia* genus are part of the skin flora, but they become pathogenic in situations of immunological imbalance. The diagnosis of pityriasis versicolor is usually clinical in cases of characteristic presentation and location. Direct mycological examination may be helpful, especially in atypical cases, evidencing the typical “spaghetti-and-meatballs” pattern, which represents pseudohyphae and fungal spores.^32^ Dermoscopy can be useful when direct mycological examination is not readily available. The differential diagnosis of pityriasis versicolor includes vitiligo, pityriasis alba, pityriasis rosea, seborrheic dermatitis, secondary syphilis, confluent and reticulated papillomatosis, and tinea corporis, among others.

Dermoscopic findings of pityriasis versicolor apparently may vary according to their clinical presentation. Hypochromic lesions usually present as well-demarcated areas with fine scaling that are located in the sulci of the skin, whereas in hyperpigmented lesions, a case report demonstrated, in addition to fine white desquamation, the presence of a pigment network consisting of brownish lines or a more homogeneous brownish pigmentation. Together, these findings may prevent unnecessary biopsies in cases of hyperchromic lesions of pityriasis versicolor.^33^ Another isolated report described the differentiation of pityriasis versicolor through dermoscopy in a patient with vitiligo. The clinical tests by Zirelli and Beznier were negative; however, on dermoscopy, it was possible to observe fine scaling along the skin creases over a hypopigmented background. The authors called this dermoscopic finding “wire fence” and reported that the finding could be used as a quick and easy way to diagnose pityriasis versicolor.^34^

**Figure 8:**
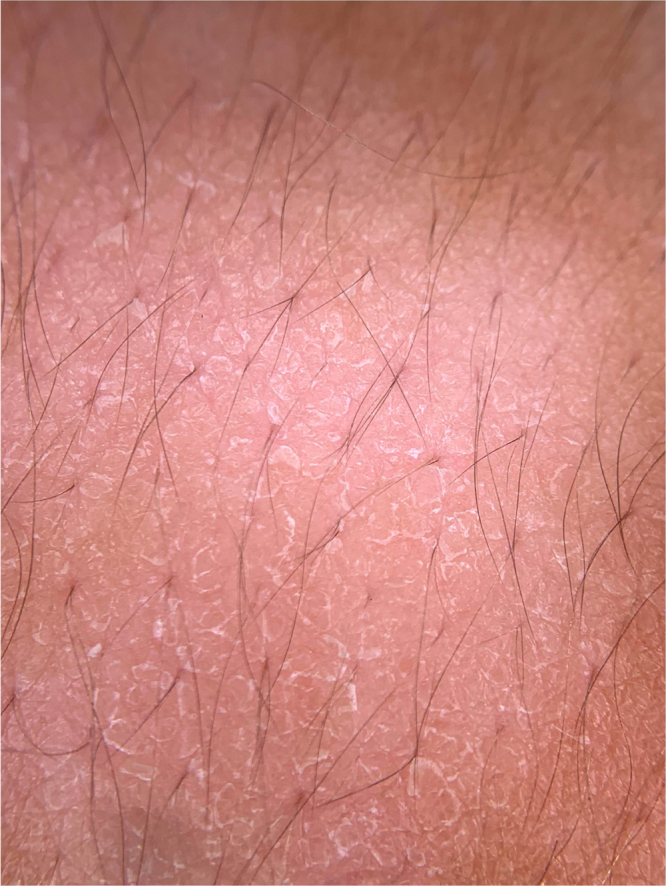
Dermoscopic image of pityriasis versicolor, demonstrating diffuse fine scaling. (FotoFinder, original magnitude ×20). Source: Authors' personal collection.

A recent series evaluated 178 pityriasis versicolor lesions in 125 patients using dermoscopy. Clinically, 164 lesions were hypopigmented and 14 were hyperpigmented.^35^ Non-homogeneous pigmentation was the most commonly observed finding in hypopigmented lesions (n = 152, 92.68%), but it was also observed in hyperpigmented ones (n = 14, 100%). Scaling was observed in 142 hypopigmented lesions (86.56%) and in 13 hyperpigmented (92.86%) (Fig. 8). Desquamative plaques were more common in hypopigmented lesions (n = 95, 57.92%), and scaling in sulci was more common on dermoscopy of hyperpigmented lesions (n = 5, 35.71%). Imperceptible ridges and sulci and perilesional hyperpigmentation were findings also observed in the study.^35^ Another series evaluated a total of 30 patients with pityriasis versicolor confirmed by clinical and mycological examination (KOH), of which 24 were hypochromic lesions, 3 were hyperchromic (10%), and the remaining 3 were both.^31^ Desquamation on the dermatoglyphs was also a relevant finding (83.3% of the cases). Additionally, a contrasting halo surrounding the lesion, called contrast halo sign, was observed in 20 cases (66.7%). In hypopigmented variants, this halo appeared as an increase in pigmentation and there was a halo of hypopigmentation in hyperpigmented lesions.^31^

## Subcutaneous and systemic mycoses

### Sporotrichosis

**Figure 9:**
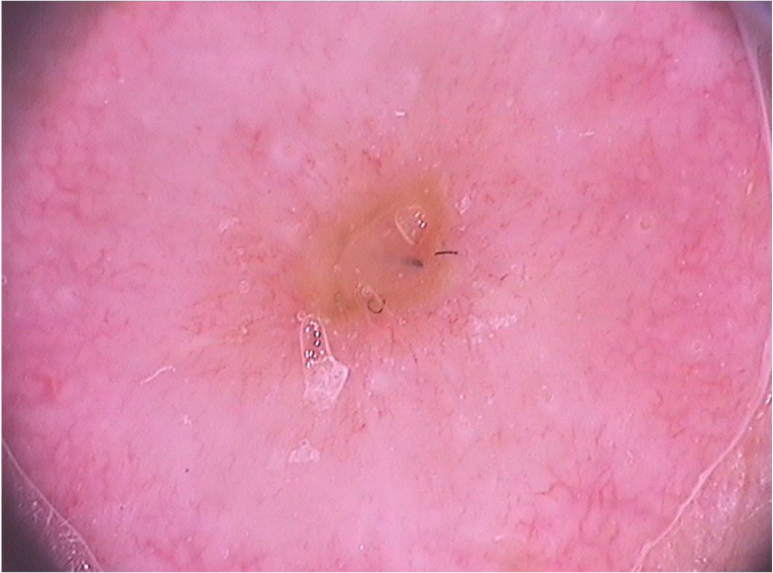
Dermoscopic image of sporotrichosis showing erythema and central areas of erosion. (FotoFinder, original magnitude ×20) Source: Collection of Hospital de Clinicas de Porto Alegre.

Sporotrichosis is a skin infection caused by the fungus *Sporothrix schenckii* and its transmission usually occurs by direct inoculation into the skin and subcutaneous tissue. In rare cases, inhalation can result in pulmonary or disseminated involvement. The most common skin manifestation is lymphocutaneous, in which verrucous papules or nodules develop at the site of inoculation, with further dissemination following the lymphatic pathways. Regarding dermoscopy, there is only one report in the literature of a patient with a disseminated condition characterized by bone and cutaneous involvement, with confluent and ulcerated erythematous papules and plaques. In this case, dermoscopic examination showed erythema, yellowish areas without structures, white-cicatricial areas, and arboriform telangiectasias. Fig. 9 shows a case with areas of erythema, linear vessels and central erosion. Additionally, some lesions had clustered pustules at their periphery.^36^ The yellowish color on dermoscopy corresponded histopathologically to areas of granulomatous inflammation, while the pustules represented neutrophilic micro-abscesses, and the white areas represented fibrosis and scar tissue.

### Chromomycosis

Chromomycosis, also known as chromoblastomycosis, is a chronic fungal infection caused by the traumatic inoculation of dematiaceous fungi, most commonly of the genus *Fonsecaea* or *Cladophialophora*. The clinical picture can vary from papular-nodular to tumoral lesions of verrucous, cicatricial, or sporotrichoid aspect.

Dermoscopy in chromomycosis shows a pinkish-white background, yellow-orange ovoid structures, polymorphic vessels, scaling, and crusts. Moreover, a frequently observed finding is reddish-black spots, which histopathologically represent transepithelial clearance of inflammatory cells, fungal debris, and minor hemorrhages.^37^ In turn, the pinkish-white background histopathologically represents areas of pseudoepitheliomatous hyperplasia and hyperkeratosis, while the yellow-orange structures correspond to areas of granulomatous inflammation. An additional case of a nodular lesion also showed a central white area with a ​​reticular aspect, which on histopathology corresponded to hypergranulosis and pseudoepitheliomatous hyperplasia.^38^ Additionally, dermoscopy can also be useful to monitor the treatment of chromomycosis, since the reddish-black spots disappear with the use of appropriate therapy.

### Cryptococcosis

Cryptococcosis is a fungal infection caused by *Cryptococcus neoformans*, being more common in immunosuppressed patients. Skin manifestations usually result from systemic dissemination, although they may rarely occur by direct inoculation. There is a wide spectrum of clinical manifestations, but the most common presentation is that of pearly white umbilicated papules, mimicking molluscum contagiosum lesions. There is only one report of the dermoscopy of cryptococcosis lesions in the literature, in a patient with acquired immunodeficiency syndrome (AIDS), neurological symptoms, and facial skin lesions.^39^ Dermoscopic examination showed structureless white areas, irregular and branched vessels, surrounded by a yellowish halo. As described above, the yellowish areas represent the granulomatous component of the lesion in histopathology, whereas the white areas correspond to fibrosis.

### Mycetoma

Eumycetoma, or mycotic mycetoma, is a chronic fungal infection that affects the skin and subcutaneous tissue. Several hyaline and dematiaceous fungal species can be the causative pathogens; however, the main ones include *Madurella mycetomatis*, *Nigrograna mackinnonii*, *Trematosphaeria grisea*, *Falciformispora senegalensis*, *Scedosporium apiospermum* and *Acremonium falciforme*. Infection typically occurs by inoculation and affects the distal portions of the lower limbs. The clinical triad is characterized by a tumor area​​, fistulous tracts, and the formation of macroscopic granules. Depending on the fungus involved, the granules can be black or yellowish-white.

As with other deep fungal infections, dermoscopy of the mycetoma also shows yellow-orange areas, corresponding histopathologically to granulomatous inflammation.^40^, ^41^ Additionally, white cicatricial areas, superficial scaling, telangiectasias, dotted vessels, and hematic crusts have also been described.^41^ Interestingly, dermoscopy can also be used for the diagnosis of granules and treatment monitoring. Litaiem et al. showed that dermoscopic examination allows the observation of black granules, which represent compact masses of dematiaceous fungi. When the granules were located more deeply, structureless bluish-white areas surrounded by a white halo and polymorphic vessels could be observed.^40^ Finally, Ankad et al. showed the alterations on dermoscopy after treatment of eumycetoma, with the reduction or disappearance of yellow-orange areas, scaling, and vascular structures and its replacement by white cicatricial areas.^41^

### Histoplasmosis

Histoplasmosis is an infection caused by the inhalation of the fungus Histoplasma capsulatum. Most infections are asymptomatic or self-limiting, but some individuals may present with severe or disseminated conditions. Skin lesions occur in disseminated histoplasmosis, and there is a wide spectrum of clinical presentations. There is only one description in the literature of the dermoscopy of skin lesions in histoplasmosis, seen in a patient with a previous history of psoriasis using an anti-TNF alpha biologic drug.^42^ On clinical examination, there was a single erythematous papule on the face in which dermoscopy showed arboriform telangiectasias and superficial desquamation, simulating a basal cell carcinoma.

### Blastomycosis

Blastomycosis is an infection caused by the inhalation of the fungus *Blastomyces dermatitidis*, which may result in an asymptomatic condition or in pulmonary and extrapulmonary manifestations, being endemic in areas of North America. The skin is the second most affected organ after the lung and usually after hematogenous dissemination, but rarely may be to traumatic inoculation. Clinically, blastomycosis causes a chronic and suppurative granulomatous reaction, which may show verrucous, ulcerated lesions or subcutaneous nodules.

Regarding dermoscopy, there are only two reports in the literature. The first case shows an erythematous-scaling nummular lesion on the face, which on dermoscopy presented papillomatous structures with a pinkish-vascular color, hematic crusts, irregular vessels, and thin scaling.^43^ Therefore, the dermoscopic pattern allowed the ruling out eczema or psoriasis. The second case reports the 25-year evolution of an ulcerative-vegetative lesion, mistakenly treated as pyoderma gangrenosum.^44^ This case also had white-pinkish papillomatous structures, hematic spots, and polymorphic vessels, including dotted, corkscrew, and serpiginous vessels.

### Leishmaniasis

Leishmaniasis is an infection caused by protozoa of the genus *Leishmania*, transmitted by the bite of phlebotomine insects (sandflies). Clinically, it is divided into cutaneous, mucocutaneous and visceral types. The disease spectrum varies according to interactions between the host, the parasite, the vector, and environmental factors. Cutaneous leishmaniasis progresses with nodular ulcerative lesions that heal, leaving atrophic areas. Laboratory diagnosis is often challenging, and an estimated 350 million people are at risk of developing the infection worldwide. The worldwide prevalence of the cutaneous form is 12 million people and the annual incidence is 1.5 million cases.^45^ Geographically, it can be divided into Old World type, which occurs in Asia, Africa and Southeastern Europe (*Leishmania donovani* complex, *Leishmania major*, *Leishmania tropica* and *Leishmania aethiopica*) and New World leishmaniasis (*Leishmania braziliensis* complex or the *Viannia* subgenus and the *Leishmania donovani* complex), that occur in Brazil and Latin American countries. This division is important, as there is clinical variation depending on the etiological agent involved: in the New World the *Viannia* complex is responsible for more prolonged and severe clinical lesions.^45^

Cutaneous leishmaniasis usually starts as an erythematous area at the site of the insect bite, which progresses to papules or nodules. After a period that can vary from two weeks to six months, lesion ulceration occurs, the edges tend to be indurated and elevated, and the center of the lesion is depressed. Additionally, some lesions may not ulcerate and remain as plaques and nodules. Lesions tend to occur in uncovered areas, especially the face and limbs. The differential diagnoses are multiple, since leishmaniasis lesions vary clinically, and include leprosy, cutaneous tuberculosis, skin cancer (including keratoacanthoma, basal cell carcinoma, squamous cell carcinoma, and amelanotic melanoma), Spitz nevus, superficial mycoses, and pyogenic granuloma, among others.^46^ In addition to clinical suspicion, the diagnosis of leishmaniasis is carried out through biopsy of the skin using different complementary methods, from anatomopathological examination to polymerase chain reaction (PCR) analysis. Dermoscopy may be helpful when there is a suspicion. The initial reports of dermoscopy on cutaneous leishmaniasis lesions were in patients with the “Old World” forms.

Its use was proposed by Lambrich et al., in a study that evaluated 26 lesions of cutaneous leishmaniasis in patients from Spain (causal agent: *L. infantum*). Generalized erythema was the most frequent finding, observed in all lesions, which corresponds to the presence of dilated vessels on histopathological examination, followed by the observation of teardrop-shaped structures called “yellowish tears” (53%), hyperkeratosis (50%), central erosion/ulceration (46%), central erosion/ulceration with hyperkeratosis (38%) and a whitish starburst pattern (38%).^46^, ^47^ The teardrop-shaped structures correspond to follicular plugs due to lateral compression of follicular openings by the growing lesion.^46^ The white starburst pattern corresponds histopathologically to hyperkeratosis and parakeratosis around the eroded area of ​​the lesion.

At least one vascular structure was observed in each lesion: comma-shaped (73%), irregular linear (57%), dotted (53%), polymorphic/atypical (26%), hairpin (19%), arboriform telangiectasias (11%), corkscrew (7%) and glomerular vessels (7%). The majority of the lesions (88%) had at least two types of vessels.^46^ The authors report that two main patterns were identified: papular lesions with a vascular component and teardrop-shaped structures, corresponding to initial lesions (observed in 26% of the lesions) and tumor lesions with erosions and ulcerations combined with hyperkeratosis, a white starburst pattern and peripheral vascular structures in more advanced lesions (46% of lesions). The authors observed a combination of these two patterns in 15% of the lesions and they observed only vessels in the remainder cases (11%).^46^

Subsequently, Yücel et al. studied 102 patients with cutaneous leishmaniasis in Turkey, confirmed by the identification of the parasite on direct microscopic examination, totaling 145 lesions (51 papules, 40 nodular-ulcerative lesions, 31 plaques, and 23 nodules). On dermoscopy, all lesions showed erythema; 58 had yellowish teardrop-shaped structures; scabs and ulcerations were seen in 51 lesions; a whitish starburst pattern in 27 lesions; salmon-colored ovoid structures in 19 lesions, and a perilesional hypopigmented halo was observed in four lesions.^48^ The presence of vascular structures was observed in 126 lesions, the most common of which was the presence of an irregular linear pattern (78 lesions), followed by an arboriform pattern (53 lesions), hairpin vessels (25 lesions), glomerular vessels (24 lesions), dotted vessels (23 lesions), comma-shaped vessels (6 lesions) and atypical polymorphic vessels (4 lesions). When dermoscopic findings were stratified according to the clinical aspect, it was observed that the yellowish teardrop-shaped structures occurred in 37% of the papules, 39% of the plaques, 61% of the nodules, and 33% of the nodular-ulcerative lesions. Ulcers and crusts were detected in 29% of plaques and in all nodular-ulcerative lesions. A starburst pattern was observed in 6% of papules, 19% of plaques, 9% of the nodules, and 40% of the nodular-ulcerative lesions. Salmon-colored ovoid structures were detected in 14% of the papules, 19% of the plaques, 17% of the nodules, and 5% of nodular-ulcerative lesions.^48^ Regarding the duration of the lesion, some findings were more frequently observed according to the time of evolution of the lesion: irregular linear, arboriform, hairpin, glomerular, dotted, comma-shaped, polymorphic and atypical linear vessels and yellowish teardrop-shaped structures, ulcers and crusts, perilesional hypopigmentation halo, and a starburst pattern were most commonly seen in lesions lasting 0 to 6 and 7 to 12 months. Salmon-colored ovoid structures were most commonly seen in lesions lasting 7 to 12, 19 to 24, and over 25 months.^48^ Milia cyst-like structures were found primarily in lesions located on the head and neck.^47^

**Figure 10:**
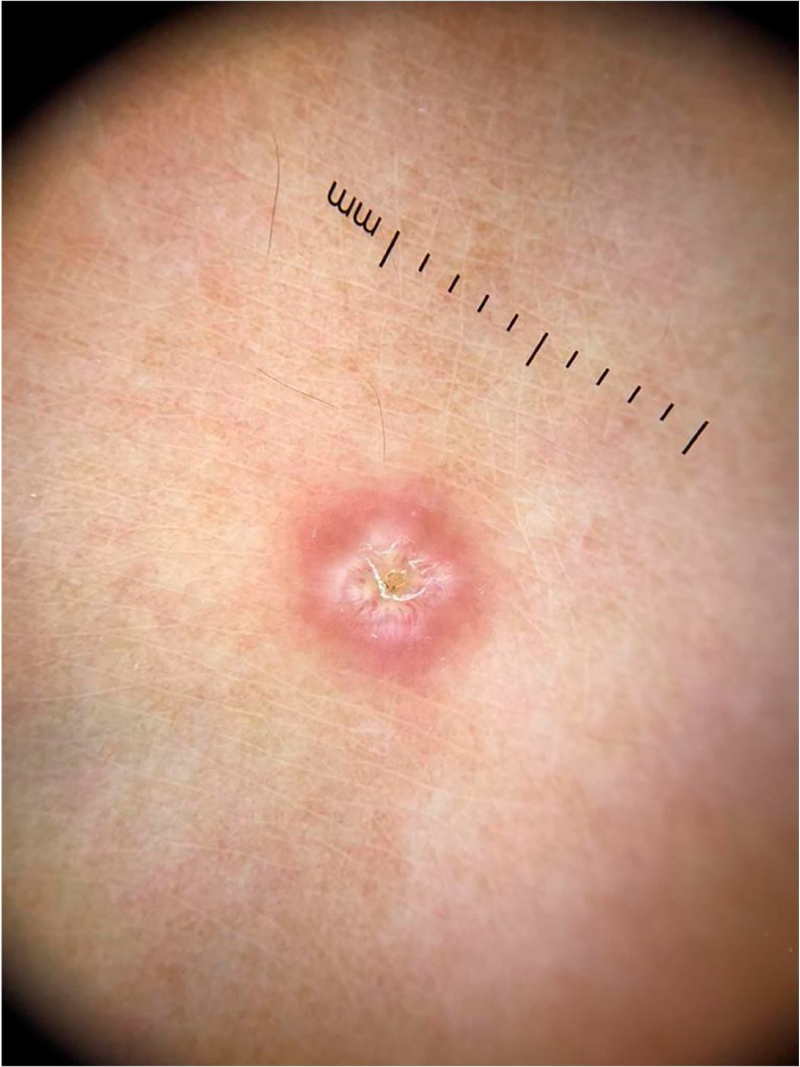
Dermoscopic image of American tegumentary leishmaniasis, showing central ulceration with homogeneous white areas and erythema with linear vessels at the periphery. (FotoFinder, original magnitude ×20). Source: Collection of Hospital de Clinicas de Porto Alegre.

Although dermoscopic findings can help in the diagnosis of cutaneous leishmaniasis, the anatomopathological examination is necessary in cases where it is not possible to exclude malignancies, such as amelanotic melanoma, especially due to the presence of structures potentially common to both, such as atypical polymorphic vessels. To date, the findings of cutaneous leishmaniases, such as the articles described above, have evaluated cases of the disease according to the species that cause leishmaniasis in the Old World. However, it is possible that there are dermoscopic differences in New World leishmaniasis. Fig. 10 shows cases of american tegumentary leishmaniasis with central ulceration, homogeneous white areas and erythema with linear vessels in the periphery.

### Arachnids

Individuals who perform recreational or professional activities in natural environments may be susceptible to contact with species of arachnids. Ticks are small representatives of this class, being in some cases, mistaken for the sudden appearance of melanocytic lesions by some patients when the parasite is small. Accidentally, they can adhere to human skin, and they can be vectors of systemic diseases. With a dermatoscope it is possible to easily identify the parasite. In general, the grayish-brown chitinous body and its legs can be seen.^49^ In one reported case, the patient had an erythematous-papular eruption in the interdigital regions of the hands, which was treated with topical corticoids and topical permethrin without success. Dermoscopic observation of the lesions allowed the identification of small dark spikes compatible with hairs of the brazilian black tarantula spider (*Grammostola pulchra*).^50^ Without the use of a dermoscope, such identification would likely be impossible, demonstrating that, even in an isolated case report, the dermoscopic pattern can be very useful for diagnostic clarification.

## Final considerations

Dermatology encompasses a huge spectrum of nosological conditions and their variants. As already demonstrated in part I of this article in dermatozoonosis and bacterial infections, dermoscopy is also very useful in the evaluation of viral and fungal skin infections, among others. It is always relevant to mention the importance of correlating dermoscopic findings with clinical history and physical examination of patients. Like any supplementary diagnostic method, it can be extremely useful in many cases and contribute little to others. This article brings up-to-date contributions to improve dermatological clinical practice through the use of dermoscopy. This is an area of ​​knowledge yet to be further explored. New descriptions of dermoscopic findings in infectious dermatoses should occur, consolidating previously described findings or making new descriptions and, ultimately, contributing increasingly more to the daily routine of dermatologists.

## Financial support

None declared.

## Authors' contributions

Renato Marchiori Bakos: Article design; article organization; drafting of the manuscript; review and approval of the final version of the manuscript.

Leandro Linhares Leite: Drafting and editing of the manuscript; review and approval of the final version of the manuscript.

Clarissa Reinehr: Drafting and editing of the manuscript; review and approval of the final version of the manuscript.

Gabriela Fortes Escobar: Drafting and editing of the manuscript; review and approval of the final version of the manuscript.

## Conflicts of interest

None declared.
